# Energy conversion, redox catalysis and generation of reactive oxygen species by respiratory complex I^☆^

**DOI:** 10.1016/j.bbabio.2015.12.009

**Published:** 2016-07

**Authors:** Judy Hirst, Maxie M. Roessler

**Affiliations:** a Medical Research Council Mitochondrial Biology Unit, Wellcome Trust/MRC Building, Cambridge Biomedical Campus, Hills Road, Cambridge, CB2 0XY, United Kingdom; b School of Biological and Chemical Sciences, Queen Mary University of London, Mile End Road, London E1 4NS, United Kingdom

**Keywords:** CW, continuous wave, ESEEM, electron spin echo envelope modulation, EPR, electron paramagnetic resonance, FeS, iron–sulfur, HYSCORE, hyperfine sub-level correlation, SMP, submitochondrial particle, SQ, semiquinone, Electron paramagnetic resonance, Iron–sulfur cluster, NADH:ubiquinone oxidoreductase, Proton-coupled electron transfer, Semiquinone, Superoxide

## Abstract

Complex I (NADH:ubiquinone oxidoreductase) is critical for respiration in mammalian mitochondria. It oxidizes NADH produced by the Krebs' tricarboxylic acid cycle and β-oxidation of fatty acids, reduces ubiquinone, and transports protons to contribute to the proton-motive force across the inner membrane. Complex I is also a significant contributor to cellular oxidative stress. In complex I, NADH oxidation by a flavin mononucleotide, followed by intramolecular electron transfer along a chain of iron–sulfur clusters, delivers electrons and energy to bound ubiquinone. Either at cluster N2 (the terminal cluster in the chain) or upon the binding/reduction/dissociation of ubiquinone/ubiquinol, energy from the redox process is captured to initiate long-range energy transfer through the complex and drive proton translocation. This review focuses on current knowledge of how the redox reaction and proton transfer are coupled, with particular emphasis on the formation and role of semiquinone intermediates in both energy transduction and reactive oxygen species production. This article is part of a Special Issue entitled Respiratory complex I, edited by Volker Zickermann and Ulrich Brandt.

## Highlights

• Current knowledge of the redox reactions catalyzed by complex I is reviewed.
• Possible quinone reduction pathways are presented.
• The presence and number of semiquinone intermediates are deliberated.
• The involvement of cluster N2/semiquinones in coupled proton transfer is discussed.
• Evidence for reactive oxygen species production by semiquinones is examined.

## 1 Introduction to complex I

Complex I (NADH:ubiquinone oxidoreductase) [1] plays a central role in the cellular metabolism of humans and many other organisms. It oxidizes NADH in the mitochondrial matrix to regenerate the NAD^+^ pool, sustaining the Krebs' tricarboxylic acid cycle and the β-oxidation of fatty acids. The two electrons from NADH oxidation are transferred through the enzyme and used to reduce ubiquinone to ubiquinol in the inner mitochondrial membrane, supplying the rest of the electron transport chain with electrons for the reduction of oxygen to water. The free energy produced by the redox reaction is captured and used to transport protons across the mitochondrial inner membrane, building the proton-motive force (Δp) that is consumed to support ATP synthesis and the import and export of metabolites and proteins to and from the mitochondrion. In addition, reactive oxygen species production by complex I is an important contributor to mitochondrial and cellular oxidative stress [2]. Complex I dysfunctions are caused by genetic, environmental, and pathological factors, and have been linked to both impaired catalytic ability and enhanced superoxide production [3]. Their molecular, mechanistic, and energetic consequences remain poorly understood, highlighting the need for improved basic knowledge of the enzyme's molecular structure and function.

Complex I from *Bos taurus* heart mitochondria is the most studied mammalian complex I, and has been adopted as a closely-related model for the human enzyme. The forty-five (known) proteins in mammalian complex I [4], [5] comprise 14 conserved ‘core’ subunits that are sufficient to catalyze energy transduction, and 31 ‘supernumerary’ subunits [6], [7]. The supernumerary subunits vary between species: they have been accumulated through evolution and surround the core complex. Some supernumerary subunits are known to have specific roles, and as a cohort they have been proposed to have roles in regulation, protection against reactive oxygen species, assembly, and stability [8]. Here, we concentrate on the mechanism of complex I, and thus on the 14 core subunits; we refer to the subunits throughout by their names in *B. taurus* (regardless of the species concerned).

**Fig. 1:**
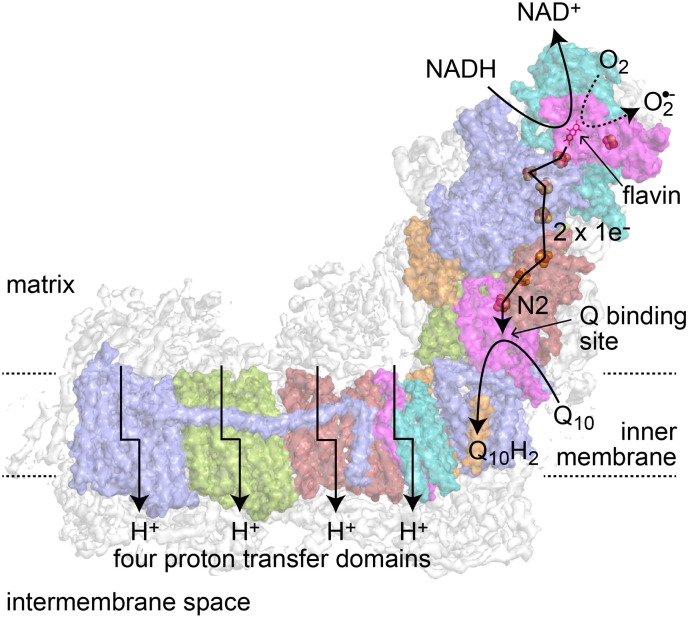
Overview of the structure and reactions catalyzed by mammalian complex I. The cryoEM density for the complete enzyme is shown in white, the fourteen subunits of the core enzyme are shown in color [5], and the reactions are indicated schematically. NADH is oxidized by a flavin mononucleotide at the top of the hydrophilic domain, then electrons are passed along a chain of iron–sulfur clusters (ending in cluster N2) to reduce bound ubiquinone. Four protons are transferred from the matrix to the intermembrane space for each NADH oxidized. The reduced flavin cofactor also reacts with molecular oxygen to form reactive oxygen species.

The core subunits form two distinct domains that are reflected in the L-shape of the complex (see Fig. 1). Seven hydrophilic core subunits (encoded by the nuclear genome) constitute the redox domain that extends into the mitochondrial matrix, and seven hydrophobic core subunits (the ND subunits encoded by the mitochondrial genome) are contained in the mitochondrial inner membrane. The structures of the core subunits were determined first in the bacterial enzymes from *Thermus thermophilus* and *Escherichia coli* [9], [10], [11], then their conserved structures were modeled using medium-resolution structural data from the mammalian enzyme from *B. taurus* [5], and the yeast enzyme from *Yarrowia lipolytica* [12].

NADH is oxidized by a non-covalently bound flavin mononucleotide at the top of the hydrophilic domain, in the 51 kDa subunit. Then a chain of seven iron–sulfur (FeS) clusters (one [2Fe–2S]^2 +/1 +^ and six [4Fe–4S]^2 +/1 +^ clusters) transfers electrons from the flavin to the quinone-binding site. An unusually-positioned additional [2Fe–2S]^2 +/1 +^ cluster is located on the opposite side of the flavin, separate from the main chain of clusters [9]; it is likely to be important for the structure around the flavin site and has no known distinct functional role [13]. The final cluster in the chain (cluster N2), which donates electrons to the bound ubiquinone substrate, is positioned more than 20 Å above the membrane surface and ~ 12 Å from the likely binding site for the ubiquinone headgroup [11]. A 30 Å long chamber connecting a narrow entry point in the membrane to this binding site has been proposed as the access route for the ubiquinone, and to accommodate its highly hydrophobic isoprenoid tail [11]. In the membrane domain, four antiporter-like structural motifs have been identified that are likely to transport one proton each per catalytic cycle. Three of these proton transfer units are formed by subunits ND2, ND4 and ND5 [10], and one by ND1, ND6 and ND4L [11]. Each of them contains two related half-channels that connect the external aqueous phase to the central membrane plane where a set of structural indicators for gated proton transfer, including charged residues and the loops of broken transmembrane helices, are located [10]. Strikingly, a long transverse α-helix runs along the membrane plane; together with supporting structure on the intermembrane face it may be involved in maintaining the integrity of the membrane domain [10]. As the transporter domains are positioned far away from the FeS cluster chain and the quinone-binding site, using the redox reaction to drive proton transport requires long-range energy transfer through the protein. The mechanism of redox-proton coupling is currently the least understood aspect of the mechanism of this huge and complicated enzyme.

In this review we focus on the redox chemistry of complex I, and particularly on current knowledge pertaining to the mechanism by which the redox energy is captured and used to initiate proton translocation. We begin with a brief overview of the ‘upstream reactions’ that deliver electrons to the coupling site (NADH oxidation and intramolecular electron transfer) then discuss the evidence for coupled chemistry at cluster N2 (the terminal cluster in the FeS chain), and the mechanism of ubiquinone reduction. In particular, we examine evidence for the involvement of semiquinone intermediates in catalysis. We close by considering how current knowledge of the redox mechanism of complex I contributes to understanding its mechanisms of energy transduction and reactive oxygen species production.

## 2 Generating the electrons for ubiquinone reduction

### 2.1 Reversible oxidation of NADH by the flavin

In mitochondrial complex I, NADH oxidation by the flavin mononucleotide is both ‘fast’ [14] and ‘reversible’ [15]. It almost certainly occurs by hydride transfer, because the highly unstable nature of the radical intermediate NAD^•^ disfavors stepwise processes and because the structure of the hydrophilic domain of *T. thermophilus* complex I with a nucleotide bound in the flavin site [16] shows the nicotinamide ring juxtaposed on the flavin ring system in an orientation consistent with a direct hydride transfer reaction [17]. The structure also revealed stacking interactions between three phenylalanine residues and the adenine ring of the nucleotide that are critical for nucleotide binding; they may help to capture the nucleotide and orientate entry of the nicotinamide ring into the cavity [18].

NADH oxidation by the flavin occurs much faster than the full catalytic cycle (proton-coupled NADH:ubiquinone oxidoreduction) of complex I can turn. Thus, it is not rate limiting in catalysis, and to study the flavin site reactions NADH oxidation must be coupled to the rapid reduction of an ‘artificial electron acceptor’ directly by the flavin so it is not controlled by the slow downstream steps of the full cycle. Kinetic studies using *B. taurus* complex I have revealed that the apparent second order rate constant for NADH binding (represented by k_cat_^NADH^/K_M_^NADH^, where k_cat_^NADH^ is the (maximum) rate of NADH oxidation observed at saturating NADH concentration and K_M_ is the Michaelis constant, equivalent to the NADH concentration required for half the maximum rate) is ~ 7.5 × 10^7^ M^− 1^ s^− 1^, approaching the diffusion-controlled limit, and that k_cat_^NADH^ (which includes both reversible hydride transfer and NAD^+^ dissociation) is greater than 15,000 s^− 1^ [14], [19]. In comparison, the maximum rate of NADH:ubiquinone oxidation that has been observed is ~ 400 s^− 1^ [20], [21].

Here, we use a thermodynamic definition of the term ‘reversible’. The fact that complex I can catalyze ‘reverse electron transfer’ (ubiquinol:NAD^+^ oxidoreduction, driven by Δp) establishes it only as an enzyme that can catalyze backwards, not as a thermodynamically-reversible catalyst that operates with a substantial rate in either direction under only minimal driving force [22]. The thermodynamic reversibility of mammalian complex I was established by varying the driving force for NADH:ubiquinone oxidoreduction to each side of the point at which it balances Δp [23], and the reversibility of NADH oxidation by the flavin, facilitated by the flavin's low reduction potential [24], was established electrochemically [15]. The rapid and reversible oxidation of NADH by complex I indicates that the oxidized and reduced states of the flavin rapidly come to redox equilibrium with the NAD^+^ and NADH in solution — a feature that was instrumental in defining the reduced flavin as the site of reactive oxygen species production (see below) [25].

**Scheme 1:**
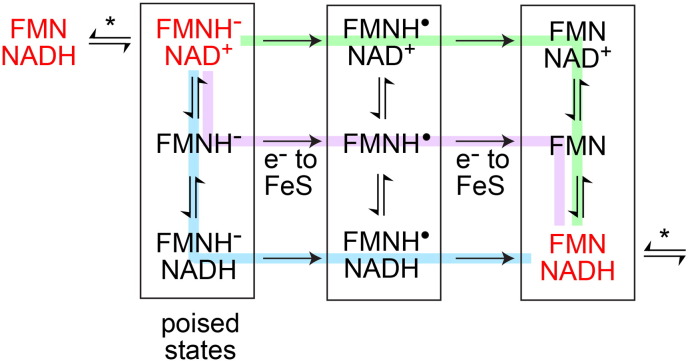
States of the flavin site during catalysis. Hydride transfer (indicated by the asterisk) occurs between the two states in red. Nucleotide binding and dissociation occur vertically, and oxidation by the FeS clusters (which occurs when ubiquinone binds to the poised enzyme) occurs horizontally. Different routes are favored in different nucleotide concentrations: green in high NAD^+^, purple in low concentrations, and cyan in high NADH.

Although knowledge of the flavin site in complex I is relatively well developed, the identities of the intermediates that dominate during catalysis (and so govern reactive oxygen species production) remain unclear. Scheme 1 shows how NADH oxidation must progress across a network of states, according to the different (as yet unknown) binding constants and nucleotide concentrations present, and identifies those ‘poised’ states in which it is likely the system waits until turnover is initiated by ubiquinone binding.

### 2.2 Intramolecular electron transfer along the FeS chain

**Fig. 2:**
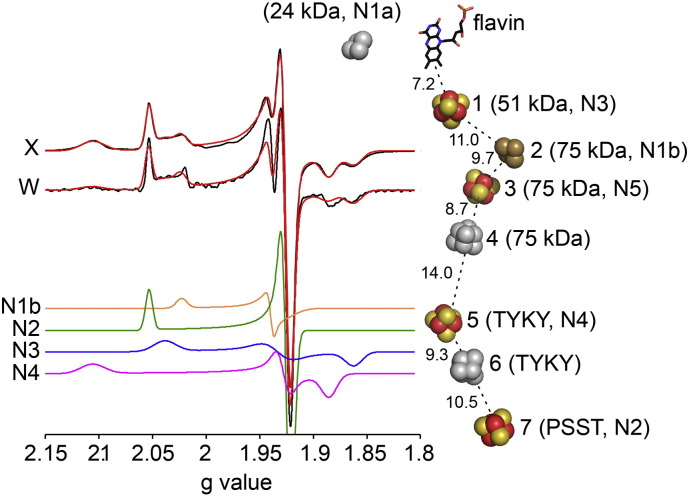
EPR spectroscopy on the FeS clusters in complex I from *B. taurus* reduced by NADH. Two spectra of complex I reduced to − 400 mV by NADH, an X-band (9.408 GHz, 12 K) CW spectrum and a W-band (93.875 GHz, 8.5 K) spectrum (black) are compared to their simulated spectra (red). The simulated subspectra of N1b, N2, N3 and N4 are presented below and the structure of the cluster chain [5] is shown on the right with the clusters labeled according to their position in the chain, their subunit, and their EPR signal. The clusters are in color if they are reduced by NADH (cluster 2 is only partly reduced) and grey if they are not; N5 is only observed at lower temperatures. EPR data taken from Roessler et al. [31].

**Scheme 2:**
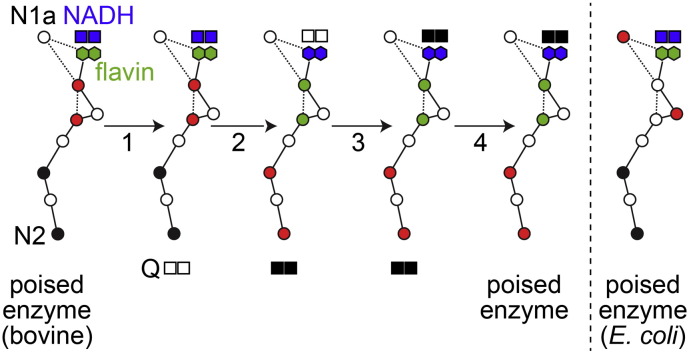
Scheme for efficient delivery of electrons to ubiquinone in mitochondrial complex I. The poised enzyme is in equilibrium with the NADH pool, with electrons occupying alternate clusters in the chain, the flavin reduced, and NADH bound. Our example refers to a fully reduced NADH pool (the NAD^+^ concentration is negligible). Step 1: ubiquinone (Q) binds to the poised enzyme. Step 2: The reduction of Q is rate limiting and so the upstream reactions (hydride transfer and intramolecular electron transfer) respond essentially instantaneously on the catalytic timescale. Step 3: NAD^+^ rapidly dissociates and is exchanged for NADH. Step 4: QH_2_ dissociates to regenerate the poised enzyme. The colors mark pairs of electrons moving through the cluster chain. The distribution of electrons in the poised *E. coli* complex I [35], [36], [45] varies from the distribution in the mitochondrial enzyme but the chain is considered similarly capable of delivering two electrons to bound Q and being rapidly back-filled by the flavin during step 2 of the reaction.

Like NADH oxidation, electron transfer along the chain of seven FeS clusters from the flavin to the quinone-binding site is generally considered fast and not rate limiting in catalysis (although there is little direct, experimental evidence for this conclusion). For an intramolecular electron transfer between two cofactors in a protein the rate is largely controlled by the distance and the reduction potential difference between the donor and the acceptor [26]. In complex I, the structure of the FeS chain (and thus each electron transfer distance) is tightly conserved in all three enzymes for which the structures have been determined [5], [11], [12]. The longest distance of 14 Å is between clusters 4 and 5, the point at which electrons move across the interface between the 75 kDa subunit in the upper part of the hydrophilic domain and the TYKY subunit in the lower part (see Fig. 2). The pattern of cluster reduction potentials along the chain is less clear. Following assignment of the electron paramagnetic resonance (EPR) signals observed in the reduced *B. taurus* enzyme [27], [28], [29] to specific structurally-defined reduced clusters [30], [31], it was possible to assign the cluster reduction potentials which had been determined in redox titrations [27], [32]. Consequently, it was noted that, in the chain in NADH-reduced *B. taurus* complex I, the electrons distribute predominantly onto alternating clusters (see Fig. 2) [31], [33]. Subsequently, Mössbauer spectroscopy confirmed that around half of the cluster cohort in mammalian complex I is reduced in the NADH-reduced enzyme [34]. Notably, although alternating reduction potentials are common features of biological redox chains [26], the same pattern is not replicated in the enzyme from *E. coli* (see Scheme 2) and so it is unlikely to be crucial for the mechanism of energy transduction.

Assuming that intramolecular electron transfer upstream of the proton-transfer-coupling point (whether it be at cluster N2 or at ubiquinone, see below) is fast and reversible denotes an enzyme that is poised to deliver two electrons rapidly to ubiquinone upon its binding — then, once the reaction has occurred, rapidly ‘backfill’ ready for the next turnover. Scheme 2 illustrates how different states of the enzyme interconvert to shuttle electrons on demand to the ubiquinone-binding site.

### 2.3 Evidence for coupled intramolecular electron transfer within the FeS chain

The reduction of the cofactors in *E. coli* complex I has been monitored by using freeze-quench strategies in combination with EPR spectroscopy [35], [36], leading to proposals that the FeS chain in complex I does not serve merely as a simple electron carrier. An additional role for the electron supply chain would not be unprecedented: the FeS relay in a class of hydrogenases (which are evolutionarily related to complex I) is able to tune the number of electrons released to the active site when the enzyme is under oxidative stress [37]. The unusual [4Fe–3S] cluster proximal to the hydrogenase active site, which bears similarity with N2 in complex I through its tandem cysteine ligation [9], is capable of using a redox-dependent conformational change to transfer two electrons to the active site and ensure that sufficient electrons are available to reduce O_2_ to water [38], [39], [40], [41]. Is it thus possible that an FeS cluster in complex I affects reactive oxygen species production by the flavin, or controls electron transfer to the quinone-binding site?

Verkhovskaya and coworkers [35] followed the development of the EPR spectrum of *E. coli* complex I upon mixing the enzyme with NADH, from 90 μs onwards. Pairs of electrons were observed to appear in the FeS chain, following the sequential oxidation of several NADH molecules, with the rate apparently limited by NAD^+^ dissociation. Intriguingly, as the chain fills with electrons the first pair of electrons appears on clusters N2 and N1a (see Fig. 2), suggesting a bifurcation event at the flavin. Although this apparent bifurcation was taken to support the previous suggestion that N1a plays a role in minimizing/preventing reactive oxygen species production by the flavin [42], the same bifurcation does not occur in the mitochondrial enzyme because cluster N1a cannot be reduced by NADH [33] (see Fig. 2). Even in the *E. coli* enzyme there is no data to support shuttling of electrons between the flavin and cluster N1a to prevent flavin radical formation during catalysis [43] and furthermore, decreasing the reduction potential of the cluster in *E. coli* so that it cannot be reduced by NADH had no effect on reactive oxygen species production [13]. It is interesting to note that in fumarate reductase the fully reduced flavin produces either H_2_O_2_ or superoxide depending on the oxidation state of a proximal [2Fe–2S] cluster [44]. This observation confirms that an FeS cluster can indeed modulate O_2_ reduction by a nearly flavin cofactor, but the cluster in fumarate reductase is part of the chain of clusters linking the two substrate-binding sites, it is not dedicated to this role. In summary, it is unlikely that cluster N1a plays any special functional role in complex I.

De Vries and coworkers also observed that the first two electrons to enter the FeS chain in *E. coli* complex I settle on clusters N2 and N1a [36]. However, based on their observation that further reduction of the chain occurs more slowly (an effect attributed to the dissociation of NAD^+^ by Verkhovskaya and coworkers) they further proposed that the redox state of N2 controls electron transfer down the chain by effecting a conformational change that decreases the rate of electron transfer between clusters 4 and 5 (see Fig. 2). The authors proposed that this feature is essential for synchronizing the electron transfer and proton transfer reactions and controlling energy transduction. However, both the proposed conformational change that propagates from cluster N2 to control the electron transfer rate, perhaps via a rearrangement of interstitial waters, and the beneficial effects of synchronizing the two reactions in this way remain to be elucidated. Finally, we note that differences in the properties of the *E. coli* and mammalian enzymes mean that all conclusions about intramolecular electron transfer drawn from the bacterial system need to be tested in the mitochondrial enzyme. Notably, the flavin site reactions in *E. coli* complex I are slower than in the *B. taurus* enzyme, cluster N1a can only be reduced by NADH in the *E. coli* complex [13], [33], [45], and the distribution of electrons in the main cluster chain is different (Scheme 2), reflecting variations in the cluster reduction potentials [27], [45]. These differences between the two systems suggest that neither fine-tuning of the cluster potentials nor a particular distribution of electrons along the chain during steady-state turnover is important for catalysis.

## 3 Is N2 involved in the coupling reaction?

**Fig. 3:**
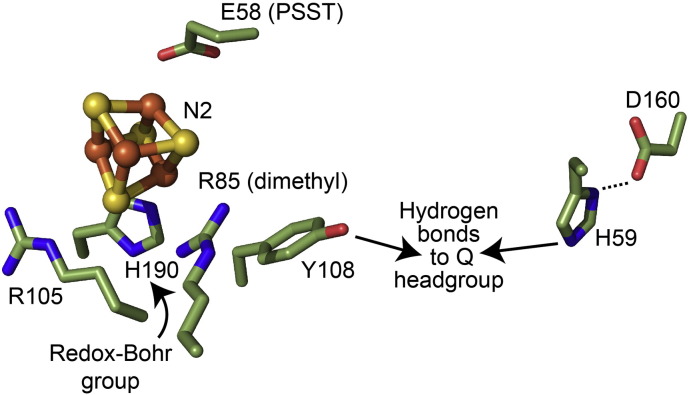
Conserved residues in the vicinity of cluster N2 and the binding site for the ubiquinone headgroup. Three conserved positively-charged residues in the 49 kDa subunit place charges close to cluster N2: R85 is dimethylated in the mammalian complex but not in *E. coli* complex I [50], and it is not conserved in *T. thermophilus*; R105 is conserved throughout; and H190 has been proposed as the redox-Bohr group that lends the reduction potential of N2 its pH dependence [53]. The carboxylate of conserved E59 in subunit PSST is approximately 9 Å from the cluster. Y108 and H59 are considered the most likely candidates to ligate the ubiquinone headgroup [11], [54], [101], although this role for H59 has recently been questioned [102]. The hydroxyl of Y108 is approximately 9 Å from the cluster and H59 is hydrogen bonded to D160; changes in the conformation of the D160 sidechain in response to ubiquinone reduction have been proposed to initiate proton translocation [103]. The figure was created using the structure of *T. thermophilus* complex I [11] with a threonine mutated to arginine to create R85, and the numbers refer to the mature proteins in *B. taurus*.

Cluster N2 is the only FeS cluster in the chain that is known to have a reduction potential that is significantly pH dependent (see Table 1) [32]. Together with the facts that N2 is both the immediate donor of electrons to the ubiquinone headgroup and (in many species) the highest potential cluster of the chain (see Table 1), this feature has long made N2 an attractive candidate for a role in proton translocation. Notably, reduced cluster N2 has not been observed in EPR spectra from *T. thermophilus* complex I (Table 1) [46], [47], indicating that it has a lower reduction potential than NADH, and the potential of N2 in *E. coli* complex I is also lower than in the other species characterized. The low potential of N2 may be an adaptation to the use of menaquinone rather than ubiquinone by *T. thermophilus* [48], and to the fact that *E. coli* switches from ubiquinone to menaquinone under anaerobic conditions [49]. The absence from *T. thermophilus* of a highly conserved Arg residue (Arg85 in the mature 49 kDa subunit of *B. taurus*) that is positioned close to N2 (see Fig. 3) may provide a partial explanation for its lower potential. Furthermore, this arginine residue has been shown to be dimethylated in the complexes from *B. taurus*, *Pichia pastoris* (a yeast), and *Paracoccus denitrificans* (a bacterium) but it is not dimethylated in *E. coli* complex I [50], providing a direct correlation between both the presence and status of Arg85 and the N2 reduction potential.

Redox-coupled changes in the ligation of cluster N2 have been proposed on the basis of changes to the electron density of reduced N2 in the hydrophilic domain of complex I from *T. thermophilus* [16]. These changes have not been verified in the intact *T. thermophilus* enzyme, or in complex I from any other species, but the idea of a cluster-core ligation change that initiates a conformational change to drive proton translocation is attractive. FeS-cluster ligation changes have been observed in other enzymes, including in the hydrogenase [4Fe–3S] cluster described above, in the [8Fe–7S] P-cluster in nitrogenases (upon formation of an amide nitrogen bond to one of the Fe subsites) [51], and in the [4Fe–4S] cluster in IspG and IspH (enzymes involved in the biosynthesis of terpenoids) in order to coordinate the substrate [52]. Whether N2 ligation changes occur as part of the catalytic cycle in complex I remains to be elucidated.

Table 1 summarizes information on the pH dependence of the N2 reduction potential. Different degrees of pH dependence have been reported in different species, but often information on the exact form of the dependence is lacking. Importantly, if the reduction potential shifts by less than 60 mV per pH unit, or only over a limited region of pH, it indicates the presence of one or more weakly-coupled protonation sites, rather than one strongly-coupled site. Because only a strongly-coupled protonation site (one that undergoes an obligatory change of protonation upon a change in N2 redox state) could be involved in coupling electron transfer to proton translocation it is important to determine the origin of the observed pH dependencies. As reviewed previously [1], mutations of both conserved and non-conserved residues in the proximity of the cluster have, to varying degrees, affected its reduction potential, its EPR spectrum, and the catalytic activity of the enzyme. Most notably, replacement of conserved His223 in the 49 kDa subunit of *Y.* *lipolytica* by methionine (His190 in *B. taurus*, see Fig. 3) led to loss of the pH dependence in the range pH 6–8, while the mutated enzyme retained its full catalytic activity [53]. Furthermore, mutation of His223 to Ala caused the EPR spectrum of N2 to be lost entirely, perhaps because its reduction potential was shifted out of range, or because a change in spin state rendered the cluster more difficult to detect — even then, the enzyme still retained some inhibitor-sensitive ubiquinone-reductase activity [54]. These findings suggest that proton-coupled electron transfer at N2 is not, in fact, involved in proton translocation. However it is perhaps premature to conclude that N2 merely serves to donate an electron to ubiquinone on the basis of applying the reduction potential measured in an equilibrium redox titration to the redox cycling of cluster N2 during catalysis. The kinetics and thermodynamics of coupled proton–electron transfer reactions observed at the [3Fe–4S] cluster in *Azotobacter vinelandii* ferredoxin I provide an illustrative example of how protons can be shuttled in and out of a hydrophobic protein interior driven by reduction and oxidation of a cluster, in this case by the swinging arm of a carboxylate residue [55]; the possibility of a similar mechanism at cluster N2, perhaps to control the transfer of protons to the ubiquinone-binding site, remains an attractive (though unsubstantiated) proposal.

Finally, complex I has been long known to be inhibited by Zn^2 +^ — but the site of inhibitory Zn^2 +^ binding is not known. EPR spectra of the mitochondrial enzyme are not affected by Zn^2 +^ [56], but Friedrich and co-workers [57] reported recently that the amplitude of the N2 EPR signal in NADH-reduced *E. coli* complex I decreases upon Zn^2 +^ binding. As N2 was found to be reduced fully by dithionite in the Zn^2 +^-bound enzyme, it is possible that Zn^2 +^ binds close to N2 and decreases its potential. Zn^2 +^ binding would normally increase a cluster’s reduction potential by an electrostatic effect, so it is possible that it prevents the coupled protonation event that causes the pH dependence. A similar decrease in potential may occur in mitochondrial complex I, but not be evident in the NADH-reduced enzyme because mitochondrial N2 has a higher potential. Zn^2 +^ binding close to N2 is also consistent with Zn^2 +^ binding preferentially to the ‘deactive state’ of the mitochondrial enzyme [56], a resting state associated with conformational changes around the quinone-binding site [58], [59]. The suggestion that Zn^2 +^ binding inhibits catalysis by interfering with coupled protonation events at cluster N2 is thus worthy of further investigation.

## 4 Evidence for semiquinone intermediates in complex I

**Scheme 3:**
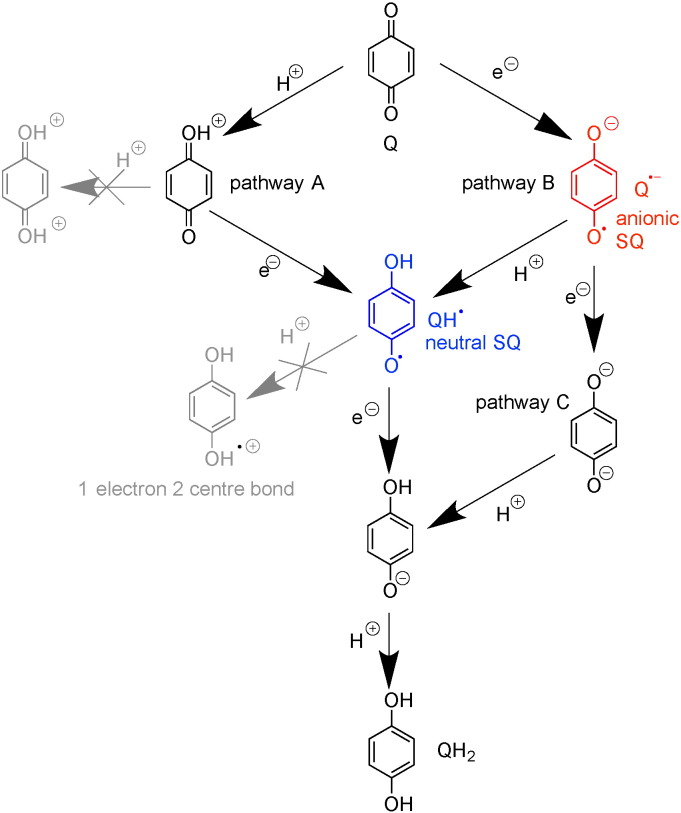
Possible pathways for the reduction of ubiquinone to ubiquinol. For simplicity, the non-redox active functional groups have been omitted. EPR-active (semiquinone) intermediates are colored. Both semiquinone intermediates have resonance forms, and are likely to interact with (unknown) neighboring groups from the protein.

With one exception [60] all the evidence for semiquinones being formed as catalytic intermediates in complex I stems from continuous-wave EPR. Until recently, most EPR studies were carried out using submitochondrial particles (SMPs), inverted membrane vesicles that contain all the enzymes of the inner mitochondrial membrane, making assigning observed semiquinone signals to the different enzymes present challenging. Semiquinone radicals were not detected in either freeze-quench study of *E. coli* complex I (with or without inhibitor, and despite the presence of ubiquinone in the samples) [35], [36]. Even in the most comprehensive studies low signal intensities have hindered detailed analyses and in fact (see Table 2) there are fundamental disagreements about most aspects of the semiquinone species that have been observed and associated with complex I including:



Before discussing these points it is first instructive to consider the possible pathways by which ubiquinone reduction may occur and semiquinone species form. Scheme 3 shows three plausible pathways, referred to as pathways A, B and C; other pathways (shown in grey in Scheme 3) are unlikely because protonation of the neutral semiquinone QH• (blue) would form a highly unstable 1-electron-2-centre bond, and because transferring two protons to ubiquinone would lead to a very high-energy intermediate carrying a double charge. Pathways A and B must involve the neutral semiquinone intermediate (blue), whereas pathways B and C involve the radical anion Q•^−^ (red). Pathway C also involves the dianion species, which (similarly to the double-protonated quinone) appears an unlikely intermediate at first sight but on whose formation a mechanism for the coupling reaction has been proposed [61]. Finally, we note that the structures shown in Scheme 3 are idealized structures and any actual intermediates formed will be affected by their protein environments. For example, an anionic semiquinone may be stabilized through hydrogen bonding, and the first intermediate of pathway A could be better represented as a hydrogen-bonded carbonyl than a protonated carbonyl. Each of the six aspects listed above is now discussed.

**Fig. 4:**
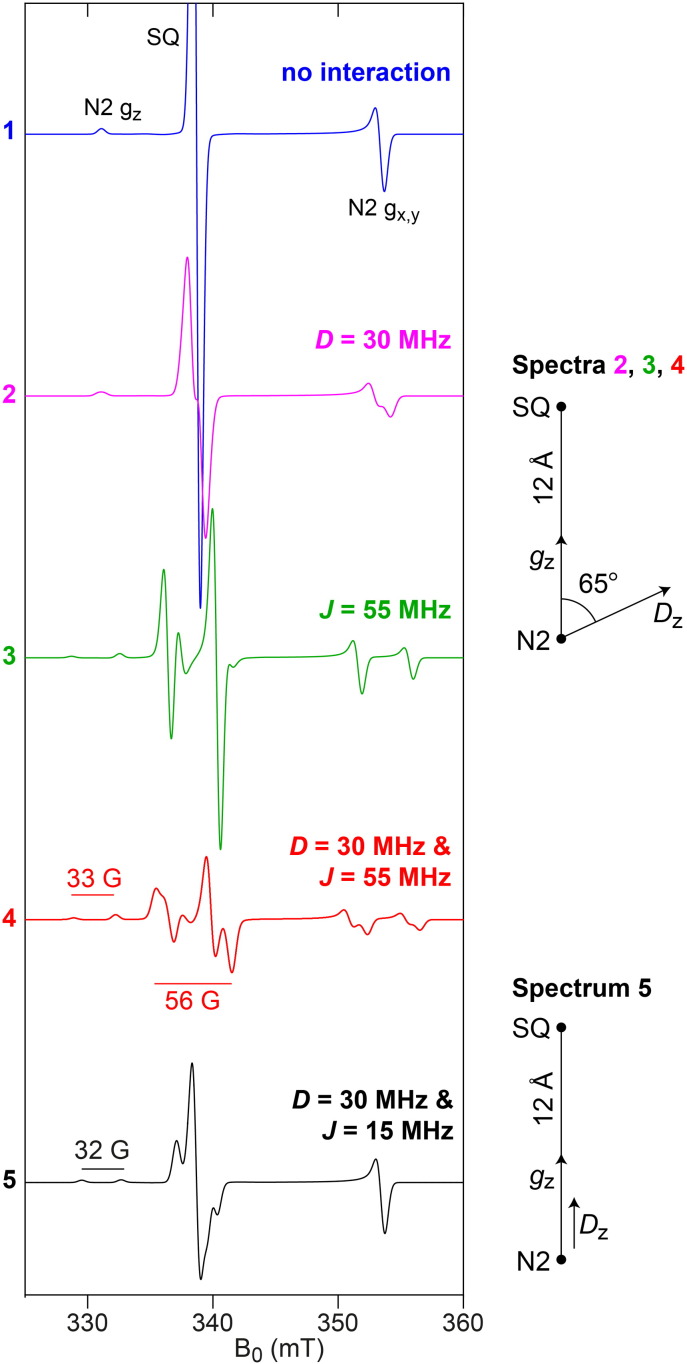
The effects of exchange and dipolar coupling on the EPR spectrum of the N2–SQ pair simulated using the parameters described by Yano et al. [71] (spectra 2–4) and using an alternative model (spectrum 5). Cluster N2 and the semiquinone radical are 12 Å apart and both treated as point dipoles. The blue trace shows the simulated spectrum from the independent (uncoupled) N2 and SQ species. For the parameters of Yano et al. the effects of the dipolar (*D*) and exchange (*J*, with *H*_exchange_ = − 2*J*(*S*_1_*S*_2_)) interactions are shown separately (magenta and green), as well as the total simulation including both *D* and *J* (red). **D** is axial with principal values [− 30, − 30, 60] MHz, with *D*_z_ at 65° relative to the N2–SQ vector. Note that we interpret the dipolar coupling of 16 MHz reported by Yano et al. to refer to the *D*_zz_ element of the **D** matrix in the tensor frame (following rotation as specified by the Euler angles, in this case β = 65°, α and γ are arbitrary); the dipolar coupling between N2 and SQ is 30 MHz (for a distance of 12 Å) and the *g* values are N2 *g*_x,y,__z_ = (1.92, 1.92, 2.05), SQ *g*_*iso*_ = 2.004. Spectrum 5 has been simulated using an alternative model in which *D*_z_ is collinear with the N2–SQ vector and *J* = 15 MHz; the dipolar coupling and *g* values are the same as in the model by Yano et al. N2 *g*_z_ is split by 90 MHz while there is no ‘net’ splitting of N2 *g*_x,y_. The microwave frequency was taken to be 9.5 GHz. Spectra were computed using the program EasySpin [104], with a common linewidth of 20 MHz.

[1]. The atomic-resolution structure of *T. thermophilus* complex I [11] revealed only one quinone binding site, and so argues against the existence of multiple locations for semiquinone species in different environments. However, the existence and detection of distinct semiquinone intermediates with significantly different properties (such as different relaxation rates, see Table 2) that are present (and can be trapped) at different points during the catalytic cycle are entirely conceivable. Furthermore, on the basis of binding large inhibitors such as amilorides to the complex, Miyoshi and co-workers [62], [63] have discussed the possibility of different entry points to a single large quinone binding site — a notion that could also explain the detection of different semiquinone intermediates if the semiquinone can adopt different positions within the site. Some EPR studies have described up to three semiquinone species (mostly by deconvoluting the data from microwave power saturation studies, see Table 2) and the number of semiquinone species described has sometimes been supported by double-integration of the signals to determine their intensities. Usually, however, these spin concentrations have been quoted relative to the intensity of the signal from cluster N2, which overlaps with other FeS signals and has very different relaxation properties to the semiquinone signals; in future, using an external standard such as CuSO_4_ for spin quantification may prove more reliable. The detection of more than one semiquinone species, and the fact that both anionic and neutral radicals have been reported (see below), may perhaps argue for pathway B. However, with so little agreement between studies and no unambiguous identification or description of the detected radicals it would be unwise to rule out pathways A and C at this stage.
[2]. Conclusions about the protonation state of complex I-bound semiquinone species observed (see Scheme 3) have so far been drawn from indirect methods, such as deuterium exchange experiments in combination with linewidths analyses [64], or from comparisons with model compounds [65]. In the absence of direct methods (see Section 4.1.), it is perhaps unsurprising that no consensus has yet been reached. Although the pH dependence of observed semiquinone signals has sometimes been reported, and indeed been used to determine the protonation state (see Table 2), higher resolution EPR methods as employed in other respiratory enzymes (see Section 4.1.) will be necessary to determine whether any radicals detected are QH• or Q•^−^ and to show how they respond to pH. This information will not only provide insights into the pathway of ubiquinone reduction (see Scheme 3), but also inform on whether bound semiquinones are able to ‘sense’ the external availability of protons.
[3]. If the reduction of ubiquinone proceeds via one or more observable semiquinone intermediates (see Scheme 3), two (likely pH dependent) reduction potentials are expected. Reduction potentials may additionally provide insights into the coupling mechanism and have been used to argue for or against particular mechanisms (see, for example, [60], [66]). However, reduction potentials refer, by definition, to an equilibrium condition that cannot be achieved while the enzyme is turning over. Few attempts have been made to determine the reduction potentials for ubiquinone bound to complex I, and the reported values differ wildly (see Table 2). Moreover, structural changes associated with quinone binding, as observed in the photosynthetic reaction center of *Rhodobacter sphaeroides* [67], can lead to redox-dependent binding modes, and the protein environment may confer very different degrees of stability on bound semiquinone species, as observed at the two quinone-binding sites of the cytochrome *bc*_1_ complex [68]. Currently, lack of consistency in the values reported in the literature precludes interpretation of the relationship between the semiquinone potentials and the energetic requirements for proton translocation in complex I.
[4]. It is undoubtedly a daunting task to extract and deconvolute *g* values, linewidths and lineshapes (Gaussian vs*.* Laurentzian), relaxation properties and the protonation states of several radical species using CW-EPR data at a single microwave frequency. The rhombicity of the semiquinone signal is not evident at X-band frequencies (~ 9 GHz), and very high microwave frequencies are required to resolve differences in the *g* values of different types of semiquinone (evident only in the 5^th^ significant figure). As an example of a case where the accurate determination of *g* values yielded mechanistic information, Stoll et al. [69] used EPR at 700 GHz to show that the *g* values of tryptophan radicals shift with hydrogen bonding. Indeed, deconvoluting X-band EPR spectra into the signals from different semiquinone species by using spectral subtraction procedures is unlikely to lead to a unique solution, especially when the linewidths are affected by saturation broadening and the total signal intensities are low. For example, Narayanan et al. [64] recently described three different semiquinone species with an estimated total concentration < 0.1 μM, corresponding to only ~ 2% of the complex I present. Furthermore, as presented in Table 2, the alternative often-used approach of deconvoluting the ‘power saturation curves’ from a set of semiquinone species is fraught with difficulties and prone to over-interpretation. Power saturation curves describe the dependence of signal intensity on microwave power; the different relaxation rates of different species cause their signals to ‘saturate’ at different microwave powers, as relaxation is outcompeted by excitation. Deconvoluting one power saturation curve by using three different species means that a single curve is fitted by nine independent parameters (describing, for each component, the homogeneity, the microwave power at which the signal has dropped to half its unsaturated intensity (*P*_1/2_), and the signal intensity as a proxy for the concentration). Importantly, power saturation curves also depend on the resonator used (owing to different power conversion factors for different resonators) and the degree of signal saturation depends on both the spin–lattice (T_1_) and spin–spin relaxation (T_2_) times, which are influenced by sample preparation conditions such as concentration, viscosity, level of oxygenation and temperature [70]. *P*_1/2_ values have not been included in Table 2 because power saturation curves describing the semiquinone species detected in complex I have been recorded under different measurement and sample conditions.
[5]. Splitting of the *g*_z_ component of the N2 signal, attributed to interaction between N2 and a nearby fast-relaxing semiquinone species under conditions of high proton-motive force, has been reported in several papers (see Table 2). However, a number of reports describing highly-coupled systems have failed to observe it (see Table 2), and, together with observation of a signal in complex I reduced to -1 V that resembles the split N2 g_z_ signal (but in the absence of ubiquinone) [33], this questions the assignment. The putative *g*_z_ splitting was used to estimate the distance between the two paramagnets as ~ 12 Å [71], based on the magnitude of the dipolar coupling (a through space interaction proportional to 1/r^3^) and by treating both the N2 cluster and the semiquinone as point dipoles (this is a rather gross approximation for the cluster, owing to the spin coupling and highly delocalized [4Fe–4S] cluster electron spin density, but before structural information became available it was the only option). For two point dipoles spaced 12 Å apart the dipolar coupling is ~ 30 MHz, and so with *D*_z_ (the unique principal axis of the **D** tensor) and *g*_z_ collinear the maximum splitting of *g*_z_ is ~ 60 MHz, smaller than the reported value of ~ 33 G (93 MHz). By including a relatively large exchange interaction of 55 MHz, and tilting the **D** tensor by 65^o^ relative to *g*_z_, Yano et al. [71] rationalized both the 33 G splitting of N2 *g*_z_ and the larger ~ 56 G apparent splitting of the SQ (semiquinone) signal. Although a large exchange coupling may be of mechanistic relevance as it indicates a facile pathway for electron transfer between N2 and bound semiquinone, the model proposed by Yano et al. requires further investigation because the complete EPR spectrum (Fig. 4, spectrum 4) simulated here (using the published parameters) does not correspond well to reported experimental spectra. Although the splitting of *g*_z_ and the width of the split ubiquinone signal are broadly reproduced by the model, the N2 *g*_x,y_ signal at higher magnetic field is clearly predicted to split — but to our knowledge a splitting in N2 *g*_x,y_ has never been observed. It is, in principle, possible that N2 *g*_z_ splits while *g*_x,y_ does not: one possible model, which made no attempt to reproduce the 56 G splitting of the quinone signal described by Yano et al., is presented in Fig. 4 (spectrum 5) and we note that the simulation resulting from this alternative model reproduces the main features in the experimental spectrum reported by Vinogradov et al. [72] quite well. In the future it will be critical to obtain and simulate complete spectra for the N2–SQ pair, preferably in the absence of other paramagnetic species. Furthermore, using the structural data now available, and by taking the spin coupling and delocalization of the electron spin density of cluster N2 into account, it should be possible to gain information on the relative orientation of N2 and SQ. A detailed picture of the electron-electron interactions would present a valuable source of mechanistic insights.
[6]. The sensitivity of SQ radical EPR signals to inhibitors and uncouplers of the proton motive force has been used both to assign observed signals to various enzymes present in SMPs, and to distinguish different SQ species. For example, a semiquinone present in complex I is expected to be abolished by rotenone (a potent complex I inhibitor), while a semiquinone present in complex II would (ideally) be unaffected. However, many studies have not been comprehensive because they did not test the effects of inhibitors of different respiratory enzymes to exclude secondary effects and cross reactions, and Table 2 shows that no consensus on how sensitive different complex I SQ species are to even rotenone has been reached. Again, one of the difficulties lies with the use of ambiguous procedures to deconvolute the contributions of multiple SQ species.

In summary, there are many unanswered questions about the presence, number, properties and roles of semiquinone intermediates formed during complex I catalysis. More detailed and quantitative analyses, in the absence of other respiratory enzymes complicating the picture, and by using methods such as pulse EPR and multiple microwave frequencies, as have been employed for the study of other respiratory enzymes, are clearly required.

### 4.1 EPR spectroscopic studies of semiquinones in other respiratory chain enzymes

Semiquinones have been investigated in detail in a number of other respiratory complexes but a comprehensive review of these studies is beyond the scope of this article. The two examples provided below serve to provide a flavor of the approaches employed to study semiquinones outside of the complex I field, with a focus on high-resolution EPR methods.

#### 4.1.1 Complex III (ubiquinol:cytochrome c oxidoreductase or cytochrome bc_1_ complex)

Structural, biochemical and biophysical characterizations of complex III considerably precede those of complex I and semiquinones in both quinone binding sites have been investigated in detail. As recently reported by Osyczka and coworkers for the complex III Q_O_ site [68], a semiquinone in the presence of a nearby reduced FeS cluster might not lead to splitting in either the SQ or FeS EPR signal. Freeze-quench experiments and careful analyses of X- and Q-band CW EPR spectra led to the discovery of an FeS–SQ_O_ spin-coupled state with a large exchange interaction. In the spin-coupled form, the semiquinone exhibits frequency-dependent *g* values and unusual properties such as a temperature dependent EPR signal that can be explained by the Leigh effect rather than the Curie law. Could some of the unusual temperature dependencies of the semiquinone signals in complex I (Table 2) be explained by such magnetic interactions? Furthermore, pulsed EPR investigations have revealed detailed information about the nuclear spins surrounding the semiquinones in complex III and identified residues involved in hydrogen-bonding [73], [74], and HYSCORE measurements of the SQ_i_ radical with ^13^C in the methoxy and methyl ring substituents, in conjunction with density functional theory, provided an estimated orientation for the substrate in the active site [75].

#### 4.1.2 Respiratory nitrate reductase

Nitrate reductase A catalyzes oxidation of quinols and reduction of nitrate to nitrite. The relatively stable menasemiquinone radical has been characterized in detail using hyperfine EPR spectroscopic methods. Using native and ^15^N labeled enzyme, Grimaldi et al. [76] used S-band (~ 3 GHz) and X-band (~ 9 GHz) ESEEM and HYSCORE to show unambiguously that the menasemiquinone is hydrogen-bonded to a nearby histidine through one of its carbonyl groups. The lower microwave frequency used brought the nitrogen coupling into an exact cancellation condition (when the nuclear Zeeman and hyperfine interactions cancel in one of the electron-spin manifolds), enabling the nuclear quadrupole parameters to be determined. Quadrupole coupling constants are very sensitive to the charge distribution around the nucleus, and the combination of the quadrupole coupling and the asymmetry parameter is a definitive characteristic with which ^14^N signals can often be assigned to specific nuclei [76]. On this basis, Grimaldi et al. were able to distinguish the histidine nitrogen from amide side-chain or peptide-bond nitrogens. Moreover, by determining the hyperfine coupling directly using ^15^N-labelled enzyme (a useful strategy that eliminates complicating quadrupolar contributions, but that is not always available), Grimaldi et al. inferred that the semiquinone is hydrogen-bonded to the observed histidine nitrogen, providing a further mechanistic clue. Subsequently, pulse X- and Q-band measurements characterized the exchangeable and non-exchangeable protons around the menasemiquinone, to produce a model for the (unusual) binding mode of the quinone substrate that may contribute to its exceptional redox properties [77].

## 5 Are reactive oxygen species produced by semiquinones in complex I?

For many years semiquinone intermediates in complex I were considered the source of reactive oxygen species production by the enzyme: the semiquinone(s) were proposed to react with O_2_ to form superoxide (O_2_•^−^) [78]. Data from isolated mitochondria (prepared predominantly from rat skeletal muscle), with superoxide produced inside the mitochondria detected outside as H_2_O_2_ [2], [79], [80], [81], [82] then substantiated this proposal. When isolated mitochondria catalyze the oxidation of ‘NADH-linked’ substrates (substrates such glutamate, malate and pyruvate that induce intramitochondrial NADH production) H_2_O_2_ production is relatively low, and addition of a complex I ‘Q-site’ inhibitor (typically rotenone or piericidin A) causes it to increase. Conversely, if the mitochondria are respiring on succinate, under conditions that promote ‘reverse-electron transfer’ (succinate:NAD^+^ oxidoreduction catalyzed by complexes I and II, supported by a large proton-motive force) then H_2_O_2_ production is relatively high, and addition of rotenone or piericidin A abolishes it. The model used to explain these observations was for one or more O_2_-reactive semiquinones bound in complex I ‘upstream’ (on the NADH-side) of the inhibitor binding site. However, in addition to the question of whether any such semiquinones exist in complex I (density from piericidin A has been detected in the binding site for the quinone headgroup in crystals of *T. thermophilus* complex I [11]), subsequent work on simpler and better characterized systems has failed to substantiate this model, leading instead to a mechanism centered on the reduced flavin cofactor.

Kussmaul and Hirst used isolated *B. taurus* complex I to show that the superoxide produced upon addition of NADH is from the fully reduced flavin, and that it occurs by a slow, second-order reaction with O_2_, with the relative concentration of reduced flavin set by a (rapid) pre-equilibrium between the flavin, NADH and NAD^+^ [25]. Subsequently, it was shown that superoxide production is blocked by high NADH concentrations, due to NADH binding in the reduced-flavin site preventing O_2_ reduction [14]. In response to suggestions that semiquinones may form and react differently with O_2_ in the isolated enzyme and in the membrane-bound enzyme, particularly because no proton motive force is present, Pryde and Hirst then recapitulated data from the isolated enzyme using complex I in SMPs, confirming the flavin-site mechanism and finding no evidence for any further, proton motive force-dependent sites of superoxide production [23].

The flavin-site model is qualitatively consistent with data from intact mitochondria, because the flavin site is upstream of the rotenone and piericidin A binding sites. During the oxidation of NADH-linked substrates by mitochondria, inhibition of complex I prevents NADH oxidation and the mitochondrial NADH/NAD^+^ pool becomes more reduced; the complex I flavin also becomes more reduced and mitochondrial H_2_O_2_ production increases. Conversely, during reverse electron transfer by mitochondria, electrons are driven into complex I from the reduced ubiquinone/ubiquinol pool, supported by a high proton motive force — so adding an inhibitor ‘cuts off’ the flavin from further reduction and H_2_O_2_ production ceases. The mechanism of superoxide production established by work on isolated complex I thus provides a firm basis for understanding superoxide production by complex I in mitochondria, and for understanding the link between mutations that cause loss of complex I activity and increased reactive oxygen species production in mitochondrial-disease patients [83].

Finally, a second route for complex I-mediated superoxide production, redox-cycling induced by the reduction of a redox-active molecule by the reduced flavin, is relevant to studies of the semiquinones formed by complex I during the reduction of hydrophilic ubiquinone substrates. Hydrophilic ubiquinones, such as ubiquinone-1 and -2 and decylubiquinone, are reduced by the complex I flavin (as well as at the ubiquinone-binding site) then reoxidized by O_2_ in solution, generating O_2_•^−^, H_2_O_2_ and semiquinone species [84]. Care must thus be taken to distinguish semiquinones formed at the flavin from the true semiquinone intermediates that may be present in the ubiquinone-binding site.

## 6 Redox intermediates in models for coupling electron transfer to proton translocation

A key question to answer about coupled electron–proton transfer in complex I is whether the reaction proceeds by a single-stroke or double-stroke mechanism. In a single-stroke mechanism a single step in the redox reaction drives all the proton transfer steps; all the ∼ 800 meV of ‘redox’ free energy is transferred to the protein at once, then all four proton transfers follow spontaneously. In a double-stroke mechanism the energy is delivered in two stages, which may or may not be equivalent. Some insights may be gained from considering the two possible sites of coupling discussed here, cluster N2 and ubiquinone. N2 is rapidly reduced by the chain, and it is predominantly reduced during steady-state catalysis, so N2 oxidation (by ubi(semi)quinone) is more likely to be coupled to proton transfer than N2 reduction. For ubiquinone, possible coupling points are ubiquinone binding, reduction of ubiquinone (either by two electrons to ubiquinol, or stepwise via the semiquinone radical), and ubiquinol dissociation.

For each redox cycle of one NADH oxidized and four protons pumped, cluster N2 must be reduced and reoxidized twice (assuming it is restricted to one electron transfers). Thus, proton translocation coupled to N2 is most consistent with a two-stroke mechanism. In the simplest model, both transitions are equivalent and they both drive two channels to each transfer one proton. The two steps in the reduction of ubiquinone may also be envisaged to drive two channels in a similar manner, as embodied in the ‘two-state stabilization-charge’ mechanism proposed by Brandt [66]. Brandt originally argued in favor of only two of the antiporter-like domains (subunits ND2 and ND5) being active in proton translocation, but the subsequent identification of a fourth antiporter-like domain for proton transfer in the structure of *T. thermophilus* complex I [11], together with additional data from site-directed mutagenesis [85], has rendered this mechanism less attractive. It is important to recognize, however, that two stroke mechanisms do not require a common two-proton transfer step to be repeated twice: the protein may store energy from the first stroke and initiate the transfer of all four protons only when the second stroke occurs: the key is that energy is provided, but not necessarily consumed, in both strokes. Indeed, the symmetry mismatch in the number of ATP molecules synthesized and the number of protons translocated by a complete turn of the two rotors in F_1_F_O_-ATP synthase provides an example of energy storage within a bioenergetic enzyme (in the mammalian enzyme each 120° turn of F_1_ is driven by 2.7 steps of 45° in the F_O_ proton-translocating ring) [86].

Nonetheless, identification of the fourth proton channel in complex I has favored proposals for single-stroke mechanisms. Verkhovsky et al. have proposed that the translocation of all four protons is driven after the reduction of the quinone (based on their suggestion that the reduction potential for the bound ubiquinone is below − 300 mV) [87], and Efremov and Sazanov proposed that all four proton transfers are coupled to the protonation of a doubly-anionic Q^2 −^ species, within a single site [61]. Both mechanisms rely on the protein environment to control both the one-electron reduction potentials for the Q/SQ and SQ/QH_2_ transitions, and proton transfer into the site. Verkhovsky et al. further proposed that the quinone species moves between two binding sites, which confer tight- and weak-binding properties upon it, a feature in common with the mechanism proposed earlier by Ohnishi [88]. However, structural data have provided no evidence for a second distinct ubiquinone binding site, and it is unclear why movement of the ubiquinone species between two sites is required to confer tight- and weak-binding properties upon it, in different states of the catalytic cycle.

To distinguish between current and future proposals for the coupling mechanism, a clear picture of the structures, thermodynamics and kinetics of the reaction intermediates formed during catalysis will be crucial. It will be important to establish, beyond any doubt, whether N2 plays a role in the coupling reaction — either directly by coupled chemistry linked to N2 redox cycling, or as part of a ‘catalytic unit’ comprising cluster N2 and bound ubiquinone. Depending on which steps are coupled to generation of the proton motive force, SQ intermediates will either be stabilized by it (for example, if proton translocation is linked to electron transfer from N2) or formed only transiently regardless of the proton motive force (for example, if proton translocation is linked to protonation of Q^2 −^). Questions to answer include: Do SQ intermediates ever accumulate to significant levels? How many different species can be identified and are they anionic or neutral? Where are they located and which residues do they interact with? What are the reduction potentials associated with SQ formation (with the caveat that reduction potentials are equilibrium measurements and so must be treated carefully when referring to turnover conditions)? When semiquinones are present, is N2 oxidized or reduced? Does N2 interact with the bound ubiquinone species, other than just as a simple electron donor? Understanding the intermediates of ubiquinone reduction by cluster N2 in complex I is a crucial part of defining the mechanism of the enzyme, and for understanding how energy provided by the redox reaction is harnessed to drive proton translocation at distant sites.

## Transparency document

Transparency document.

## Figures and Tables

**Table 1:** Reduction potentials of cluster N2 in complex I from different species, and the dependence of the potentials on pH

Species	E_m_ (mV) pH 7	pH dependence	References	Preparation
*B. taurus*	− 140	− 60 mV/pH	[32]	Submitochondrial particles & mitochondria
− 154 at pH 7.8	Not determined	[89]	Isolated complex I
Pigeon heart	− 20	− 60 mV/pH	[32]	Submitochondrial particles & mitochondria
*Y. lipolytica*	WT: − 140 H226M: − 220	WT: − 36 mV/pH H226M: none (pH 6–8)	[53]	Mitochondrial membranes
*E. coli*	− 220	Yes	[45]	Isolated enzyme
~− 200/− 300 (biphasic titration curve)	Not determined	[90]	Isolated enzyme
*R. sphaeroides*	~− 90	Yes	[91]	Isolated enzyme (modified N2 signal)
*P. denitrificans*	− 124	~− 60 mV/pH	[92]	Membrane particles
*T. thermophilus*	N2 not observed	Not determined	[46], [47]	Membrane particles & enzyme hydrophilic domain

**Table 2:**
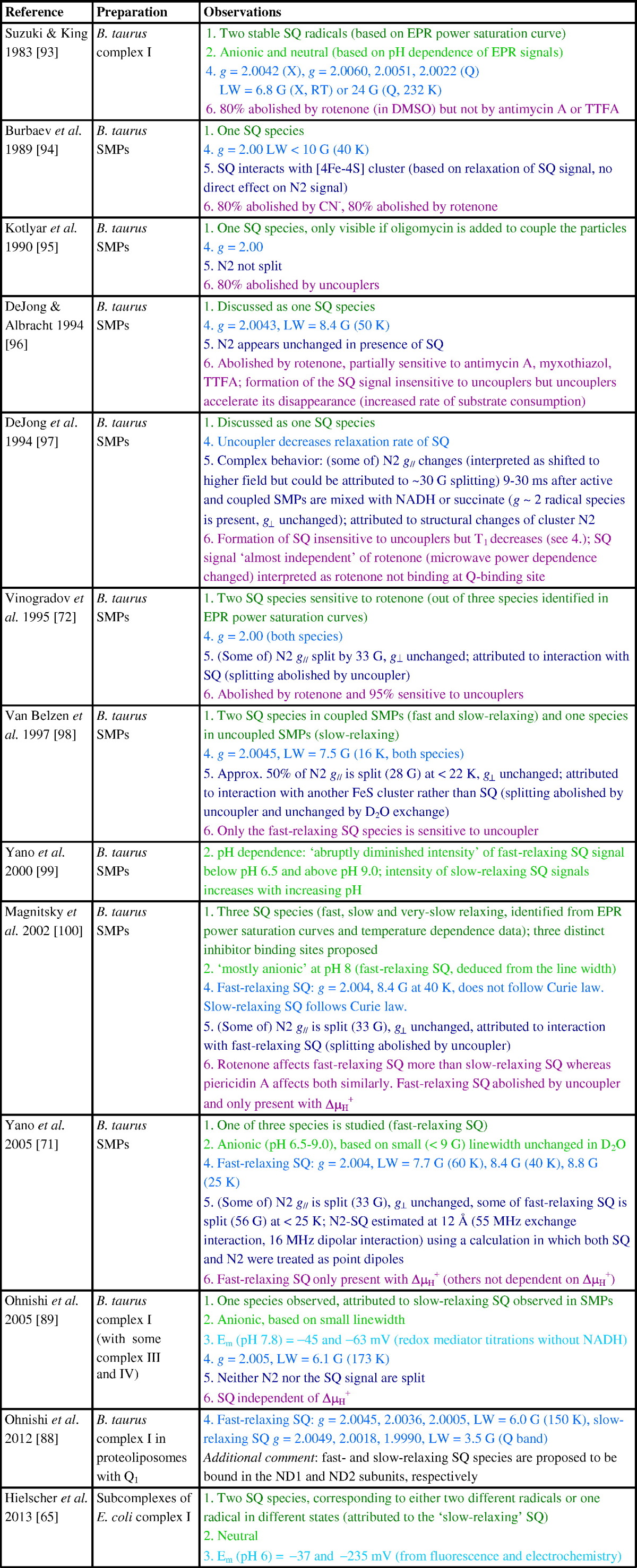

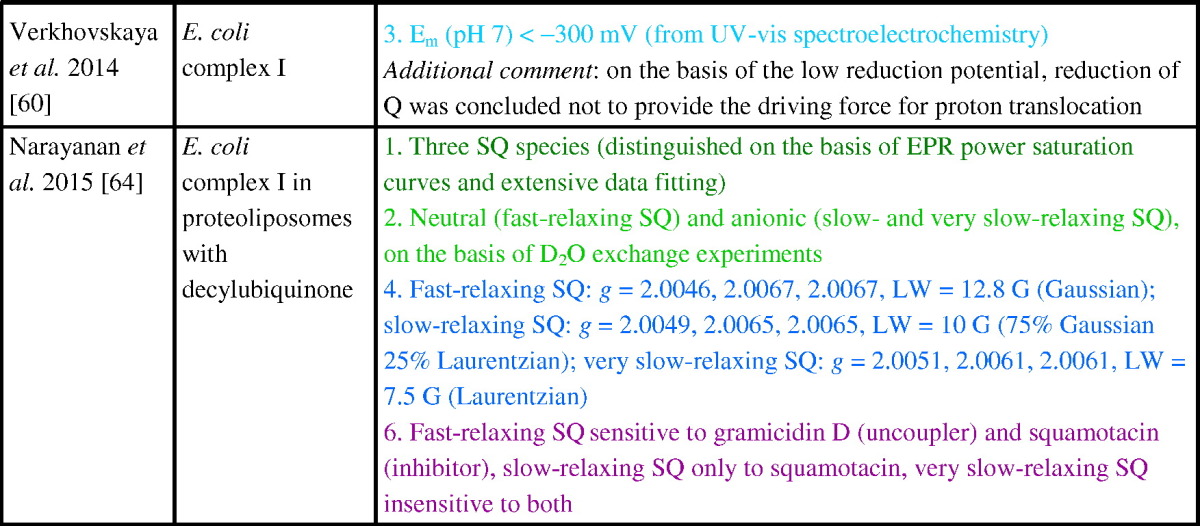
Summary of observations on semiquinone species reported in the literature

EPR parameters were obtained at X-band (~ 9 GHz) unless otherwise indicated. Q indicates measurements taken at Q-band (~ 35 GHz).

LW: line-width; RT: room temperature.

Inhibitors: rotenone and squamotacin (complex I), TTFA (2-thenoyltrifluoroacetone, complex II), antimycin A and myxothiazol (complex III), CN (cyanide, complex IV), oligomycin (complex V).
